# Characterization of 14 *Triticum* species for the *NAM-B1* gene and its associated traits

**DOI:** 10.1371/journal.pone.0287798

**Published:** 2023-08-22

**Authors:** Fatemeh Shoormij, Aghafakhr Mirlohi, David Chan-Rodriguez, Hanna Bolibok-Brągoszewska, Ghodratollah Saeidi

**Affiliations:** 1 Department of Agronomy and Plant Breeding, College of Agriculture, Isfahan University of Technology, Isfahan, Iran; 2 Department of Plant Genetics, Breeding and Biotechnology, Institute of Biology, Warsaw University of Life Sciences-SGGW, Warsaw, Poland; Government College University Faisalabad, PAKISTAN

## Abstract

**Background:**

Wheat grain protein, zinc (Zn), and iron (Fe) content are important wheat qualities crucial for human nutrition and health worldwide. Increasing these three components simultaneously in wheat grains by a single gene came into the picture through *NAM-B1* cloning. *NAM-B1* gene and its association with the mentioned grain quality traits have been primarily studied in common and durum wheat and their progenitors *T*. *dicoccum* and *T*. *dicoccoides*.

**Method:**

In the present study, for the first time, 38 wheat accessions comprising ten hexaploids from five species and 28 tetraploids from nine species were evaluated in the field for two consecutive years. Additionally, the 582 first nucleotides of the *NAM-B1* gene were examined.

**Result:**

The *NAM-B1* gene was present in 21 tetraploids and five hexaploid accessions. Seven tetraploid accessions contained the wild-type allele (five *T*. *dicoccum*, one *T*. *dicoccoides*, and one *T*. *ispahanicum*) and fourteen the mutated allele with a ‘T’ insertion at position 11 in the open reading frame, causing a frameshift. In hexaploid wheat comprising the gene, only one accession of *T*. *spelta* contained the wild-type allele, and the rest resembled the insertion mutated type. In the two-year field experiment, eight accessions with the wild-type *NAM-B1* allele had significantly higher protein, Zn and Fe grain content when compared to indel-type accessions. Additionally, these accessions exhibited a lower mean for seed-filling duration than all other accessions containing indel-type alleles. In terms of grain yield, 1,000-kernel weight, kernel diameter, and kernel length, *T*. *dicoccum* accessions having wild-type alleles were similar to the indel-type accessions over two years of evaluation.

**Conclusion:**

These findings further support the possibility of simultaneous improvement of wheat grain protein, Zn, and Fe content by a single gene crucial for human nutrition and health worldwide.

## Background

A large proportion of the world’s population relies on wheat to provide plant protein and nutrients, as well as micronutrients, such as iron (Fe) and zinc (Zn), for their daily diets [1]. Wheat domestication and significant advances in plant breeding focusing on yield improvement have sacrificed quality traits like grain protein content (GPC), Zn and Fe concentration [2, 3]. With the growing world population and widespread food shortages, predominantly in developing countries, the wild relatives of domesticated wheat containing broader genetic diversity and higher concentrations of many grain nutrients than modern wheat cultivars have gained attention [4]. This indicates that genetic improvement of these traits in contemporary wheat cultivars is possible if reliable genetic information from related species of cultivated wheat is available [5, 6]. High grain protein content and micronutrient concentration are among the most critical factors determining wheat nutritional and end-use quality [7]. The genetic basis of these traits in wheat has been a continuous focus for researchers to improve grain wheat quality. In search of genes for high grain protein content, Avivi [8] identified an allele for high GPC in wild emmer wheat (*Triticum turgidum* ssp. *dicoccoides* Körn. ex Asch. and Graebn.) Thell. (FA15-3), the ancestor of cultivated pasta wheat (*Triticum turgidum* var. *durum* (Desf.) Husn.). Analyses of substitution lines of the chromosomes of the wild emmer in durum cultivar Langdon [9] and of a population of recombinant substitution lines [10] identified a QTL on the proximal region of the short arm of chromosome 6B. The *NAM-B1* was later found to be a simple Mendelian locus within this QTL [11–13]. The *NAM-B1* was positionally cloned by Uauy et al. [14] and was shown to encode a NAC transcription factor *NO APICAL MERISTEM-B1* (*NAM-B1*) that accelerates senescence and increases nutrient remobilization from leaves to developing grains. The reduced transcript level of *NAM-B1* using RNA interference (RNAi) caused a significant reduction of more than 30% in GPC, 36% in Zn, and 38% in Fe content in wheat grain [14]. Further research confirmed the significant effect of *NAM-B1* on wheat GPC, and grain micronutrients [7]. Using recombinant chromosome substitution lines (RSLs), constructed for genetic and physical mapping of *NAM-B1*, it was found that RSLs carrying the functional *NAM-B1* allele contained on average 12% higher Zn, 18% higher Fe, 29% higher Mn, and 38% protein content than the RSLs carrying the nonfunctional allele from cultivated durum wheat [15]. A comparison of near-isogenic lines (NILs) with contrasting *NAM-B1* alleles in tetraploid and hexaploid wheat showed that functional *NAM-B1* considerably boosts protein yield and content in grain [16]. Eagles et al. [17] used near-isogenic lines with and without the *NAM-B1* gene in three Australian-adapted genetic backgrounds and displayed that *NAM-B1* increased GPC and reduced grain weight, with a negligible effect on grain yield. The effect of the functional *NAM-B1* allele on GPC was found to be very stable in different environments [9, 18, 19]. The non-functional allele contains a ‘T’ insertion at position 11 in the open reading frame, causing a frameshift. Uauy et al. [14] reported the presence of the wild-type *NAM-B1* allele in all 42 wild emmer accessions and 17 of the 19 domesticated emmer accessions examined. They also showed that the 57 durum lines and 34 hexaploid wheat varieties representing different market classes and geographic locations tested contained only the mutated allele. Subsequently, an enormous number of wheat varieties were investigated for *NAM-B1*, mostly belonging to four categories of wild emmer, domesticated emmer, durum, and bread wheat [20–24]. In these studies, the functional wild-type *NAM-B1* allele was most prevalent in wild and domesticated emmer, the mutated non-functional allele in durum, and the deletion type in bread wheat. The impact of functional *NAM-B1* on grain quality and yield traits has been studied mostly in durum and bread wheat. Also, under different environments, its consistent positive effects on grain protein, Fe, and Zn content were proved with slightly negative impacts on yield [16, 18, 19]. It has also been shown that *NAM-B1* plays an important role in plant defense and stress responses [25, 26], which makes it even more relevant. Since it is less examined in a broad range of wheat species, the present study aimed to characterize a wide range of wheat species for *NAM-B1* alleles through sequencing and field evaluation for associated traits in two consecutive years.

## Results

### Distribution of *NAM-B1* allele in different wheat species

The 38 wheat accessions used in this study consisted of 28 tetraploids from nine species and ten hexaploids from five species. The *NAM-B1* gene was present in 21 tetraploids (72.41%) and five hexaploid accessions (50%). Among the 21 tetraploids containing this gene, seven represented the wild-type allele, and the rest had the insertion mutated type allele. In the five hexaploid accessions with the gene, only one *Triticum aestivum* L. subsp. *spelta* (L.) Thell. (#29) accession contained the wild-type allele, and the rest resembled the insertion mutated type. Interestingly, five of the seven tetraploid accessions with the wild-type alleles belonged to *Triticum turgidum* L. subsp. *dicoccum* (Schrank ex Schübl.) Thell., one to *T*. *dicoccoides*, and one to *Triticum ispahanicum* Heslot (#3–7, #1, and #2 in Table 1, respectively). The nine durum accessions contained only the insertion mutated type allele. The other five tetraploid wheat species, including *Triticum turgidum*, *Triticum aethiopicum* Jakubz., *Triticum turgidum* L. subsp. *polonicum* (L.) Thell., *Triticum turgidum* L. subsp. *carthlicum* (*T*. *persicum)* (Nevski) Á. & D. Löve, and *Triticum turgidum* L. subsp. *turanicum* (Jakubz.) Á. & D. Löve, each with two accessions, showed insertion mutated type allele in one accession and the deletion type in the other (Table 1). Hexaploid species *Triticum vavilovii* (Tumanian) Jakubz. (#31), *Triticum petropavlovskyi* Udachin and Migush (#37), and two *synthetic crosses* (#34 and 35) had the insertion mutated type allele and *T*. *spelta* (#30), *Triticum aestivum* (#33, 34), *Triticum Compactum* Host (#36), and *Triticum aestivum* L. subsp. *sphaerococcum* (Percival) Mackey (#38) did not contain the *NAM-B1* gene (Table 1).

**Table 1:** Mean comparison of different traits for 38 wheat accessions from various species in each year of evaluation (2018–2020) and their *NAM-B1* allele status.

No.	Species	PL^δ^	*NAM-B1*	SNP^¥^	GPC^-1yr^	GPC^-2yr^		Zn^-1yr^	Zn^-2yr^		Fe^-1yr^	Fe^-2yr^		SFD^-1yr^	SFD^-2yr^	
1	*T*. *dicoccoides*	4x	Wild type	C	16.40^a^^ζ^	15.36^b^	15.88^£^	77.28^b-e^	83.84^ab^	80.56^£^	77.28^bc^	94.68^a^	85.98^£^	21.50^m^	9^j^	15.25^£^
2	*T*. *ispahanicum*	4x	Wild type	C	14.88^bcd^	14.86^bc^	14.87	84.77^ab^	70.99^b-h^	77.88	85.09^a-e^	82.47^a-e^	83.78	25.50^l^	12.50^i-j^	19
3	*T*. *dicoccum*	4x	Wild type	C	15.62^ab^	14.40^b-e^		71.72^c-f^	90.02^a^		81.71^a-g^	79.57^a-f^		26^l^	13^i^	
4	*T*. *dicoccum*	4x	Wild type	C	15.14^abc^	14.40^b-e^		76.91^bcd^	73.99^b-f^		81.43^a-g^	79.90^a-f^		24^lm^	14^hi^	
5	*T*. *dicoccum*	4x	Wild type	C	15^bc^	14.21^c-f^		73.52^b-d^	77.37^a-d^		74.23^a-k^	81.99^a-e^		24.50^lm^	12.50^ij^	
6	*T*. *dicoccum*	4x	Wild type	C	14.89^bcd^	14.85^bcd^		71.48^c-g^	75.26^b-e^		73.85^a-k^	83.84^abc^		25^l^	13.50^i^	
7	*T*. *dicoccum*	4x	Wild type	C	14.82^bcd^	16.92^a^	15.1	93.51^a^	80.25^ab^	78.40	88.27^abc^	90.04^ab^	81.48	26.50^kl^	13.50^i^	15.45
8	*T*. *dicoccum*	4x	Absent	-	13.55^d-j^	13.45^e-i^		57.91^e-k^	66.46^c-i^		62.76^e-n^	64.13^f-l^		32.50 ^e-i^	19^efg^	
9	*T*. *dicoccum*	4x	Absent	-	13.35^e-j^	13.48^e-i^	13.45	70.88^b-f^	80.28^ab^	68.88	74.65^a-k^	82.46^a-e^	71	32.50^e-i^	19^efg^	25.75
10	*T*. *durum*	4x	Mutated	T	13.06^f-j^	12.05^l-o^		57.30^f-k^	64.24^d-k^		53.33^j-n^	50.47^kl^		33.50^c-g^	20^d-g^	
11	*T*. *durum*	4x	Mutated	T	12.22^jk^	12.43^j-o^		58.13^e-k^	57.20^i-n^		46.16^n^	61.21^g-l^		31.50^f-j^	23^a-d^	
12	*T*. *durum*	4x	Mutated	T	12.40^jk^	12.17^k-o^		57.75^f-l^	59.79^g-n^		48.82^mn^	58.54^h-l^		33^d-h^	20.50^c-g^	
13	*T*. *durum*	4x	Mutated	T	13.39^e-j^	12.69^i-n^		64.57^e-k^	62.82^e-l^		55.27 ^i-n^	60.10^g-l^		33^d-h^	21.50^c-f^	
14	*T*. *durum*	4x	Mutated	T	13.06^f-j^	12.57^i-o^		65.23^e-j^	58.31^h-n^		57.79^i-n^	56.89^i-l^		36^bc^	22^b-f^	
15	*T*. *durum*	4x	Mutated	T	12.90^g-j^	12.41^j-o^		59.74^h-l^	54.16^i-n^		51.13^lmn^	61.58^g-l^		31.50^g-j^	21.50^c-f^	
16	*T*. *durum*	4x	Mutated	T	13.20^e-j^	12.26^j-o^		61.14^f-l^	61.35^f-n^		52.46^k-n^	49.95^l^		35.50 ^bcd^	20.50 ^c-j^	
17	*T*. *durum*	4x	Mutated	T	13.09 ^d-h^	12.90^h-m^		52.81^k-l^	62.27^e-m^		48.35^n^	54.17^jkl^		33^d-h^	23^a-d^	
18	*T*. *durum*	4x	Mutated	T	12.40^jk^	12.53^i-o^	12.65	59.99^e-k^	53.05^i-n^	59.40	52.17^k-n^	59.93^g-l^	54.35	34^c-f^	21 ^c-g^	27.44
19	*T*. *turgidum*	4x	Absent	-	12.47^jk^	12.30^j-o^		50.47^j-k^	55.89^i-n^		65.17^d-n^	75.46^b-f^		30^ij^	20^d-g^	
20	*T*. *turgidum*	4x	Mutated	T	14.02^c-g^	12.39^j-o^	12.38	64.59^e-j^	59.03^g-n^	57.49	77.28^a-i^	73.81^c-h^	72.93	30.50^hij^	21.50^c-f^	25.50
21	*T*. *aethiopicum*	4x	Absent	-	13.35^e-j^	12.92^h-m^		55.57^kl^	62.30^e-n^		57.87^h-n^	67.19^d-j^		35.50^bcd^	26.50^a^	
22	*T*. *aethiopicum*	4x	Mutated	T	14.33^b-f^	12.70^i-n^	13.13	51.21^jkl^	55.47^i-n^	56.13	60.08^f-n^	68.55^c-j^	63.42	33^d-h^	25.50^ab^	30.12
23	*T*. *polonicum*	4x	Mutated	T	13.94^c-h^	13.02^h-m^		63.38^e-k^	80.10^abc^		87.58^a-d^	71.55^c-i^		50^a^	20^d-g^	
24	*T*. *polonicum*	4x	Absent	-	13.80^c-i^	14.13^c-g^	13.72	57.59 ^i-l^	63.96^d-k^	66.25	68.11^b-n^	50.91^kl^	69.53	31^g-j^	18.50^fg^	29.87
25	*T*. *persicum*	4x	Mutated	T	13.32^e-j^	13.74^d-h^		50.76^l^	64.07^d-k^		78.13^a-i^	64.44^d-j^		29^jk^	21^c-g^	
26	*T*. *persicum*	4x	Absent	-	13.01^f-j^	13.15^g-k^	13.30	60.66^f-l^	47.86^n^	55.83	61.91^f-n^	63.07^g-l^	66.88	30^ij^	20^d-g^	25
27	*T*. *turanicum*	4x	Mutated	T	14.16^c-g^	13.23^f-j^		68.06^c-i^	64.13^d-k^		68.20^b-n^	59.22^h-l^		33^d-h^	19.50^d-j^	
28	*T*. *turanicum*	4x	Absent	-	12.38^jk^	12.03^mno^	12.96	52.59^kl^	49.89^lmn^	58.66	59.07^g-n^	63.10^g-l^	62.39	31.50^f-j^	19.50^d-g^	25.87
29	*T*. *spelta*	6x	Wild type	C	14.84^bcd^	14.23^c-f^	14.53	76.73^bcd^	72.53^b-g^	74.63	76.40^a-i^	79.27^a-f^	77.83	26^l^	14^hi^	20
30	*T*. *spelta*	6x	Absent	-	13.24^e-j^	13.06^h-l^	13.15	64.95^e-j^	63.91^e-l^	64.43	67.17^c-n^	83.06^a-d^	75.11	37.50^b^	20^d-g^	28.75
31	*T*. *vavilovii*	6x	Mutated	T	14.12^c-g^	13.36^e-j^	13.74	76.67^bcd^	71.66^b-h^	74.16	75.24^a-j^	68.66^c-j^	71.95	38^b^	17.50^gh^	27.75
32	*T*. *aestivum*	6x	Absent	-	12.28^jk^	11.73^no^		58.96^i-l^	52.36^k-n^		56.15^i-n^	50.39^kl^		32.50^e-i^	22.50^be^	
33	*T*. *aestivum*	6x	Absent	-	12.44^ijk^	11.60°	12.01	61.19^f-l^	56.61^j-n^	57.28	60.38^f-n^	67.13^d-j^	58.51	37.50^b^	22.50^b-e^	28.75
34	*synthetic cross*	6x	Mutated	T	14.10^c-g^	13.11^h-k^		62.98^e-k^	58.12^h-n^		70.60^a-m^	66.49^e-k^		34^c-f^	24^abc^	
35	*synthetic cross*	6x	Mutated	T	12.62^h-k^	11.83^no^	12.91	58.80^i-l^	50.04^lmn^	57.48	71.16^a-m^	67.74^c-j^	68.99	34.50 ^c-f^	23^a-d^	28.87
36	*T*. *compactum*	6x	Absent	-	11.47^k^	12.38^j-o^	11.92	61.19^f-l^	48.92^mn^	55.05	65.79^c-n^	60.21^g-l^	63	30.50^hij^	19^efg^	24.75
37	*T*. *petropavlovskyi*	6x	Mutated	T	14.51^b-e^	14.15^c-g^	14.33	77.59^bc^	64.75^d-k^	71.17	71.93^a-m^	81.34^a-e^	76.63	30^ij^	19^efg^	24.50
38	*T*. *sphaerococcum*	6x	Absent	-	14.01^c-g^	12.73^h-n^	13.19	63.33^e-k^	66.23^d-j^	64.78	73.39^a-m^	70.16^c-j^	71.77	31.50^f-j^	23^a-d^	27.25
	LSD (0.05)				1.36	1.01		11.66	13.70		22.52	16.17		2.61	3.60	

GPC, Grain protein content (%), Zn, Grain zinc content (μg/g); Fe, Grain iron content (μg/g); SFD, Seed-filling duration; PL, Ploidy level.

^¥^, SNP, Single nucleotide polymorphism at position +11

^£^, Average of two years for GPC, Zn, and Fe.

^ζ^, For each column, means followed by the same letter are not significantly different, using the LSD test at a 5% probability level.

### Association between *NAM-B1* with related traits

**Fig 1:**
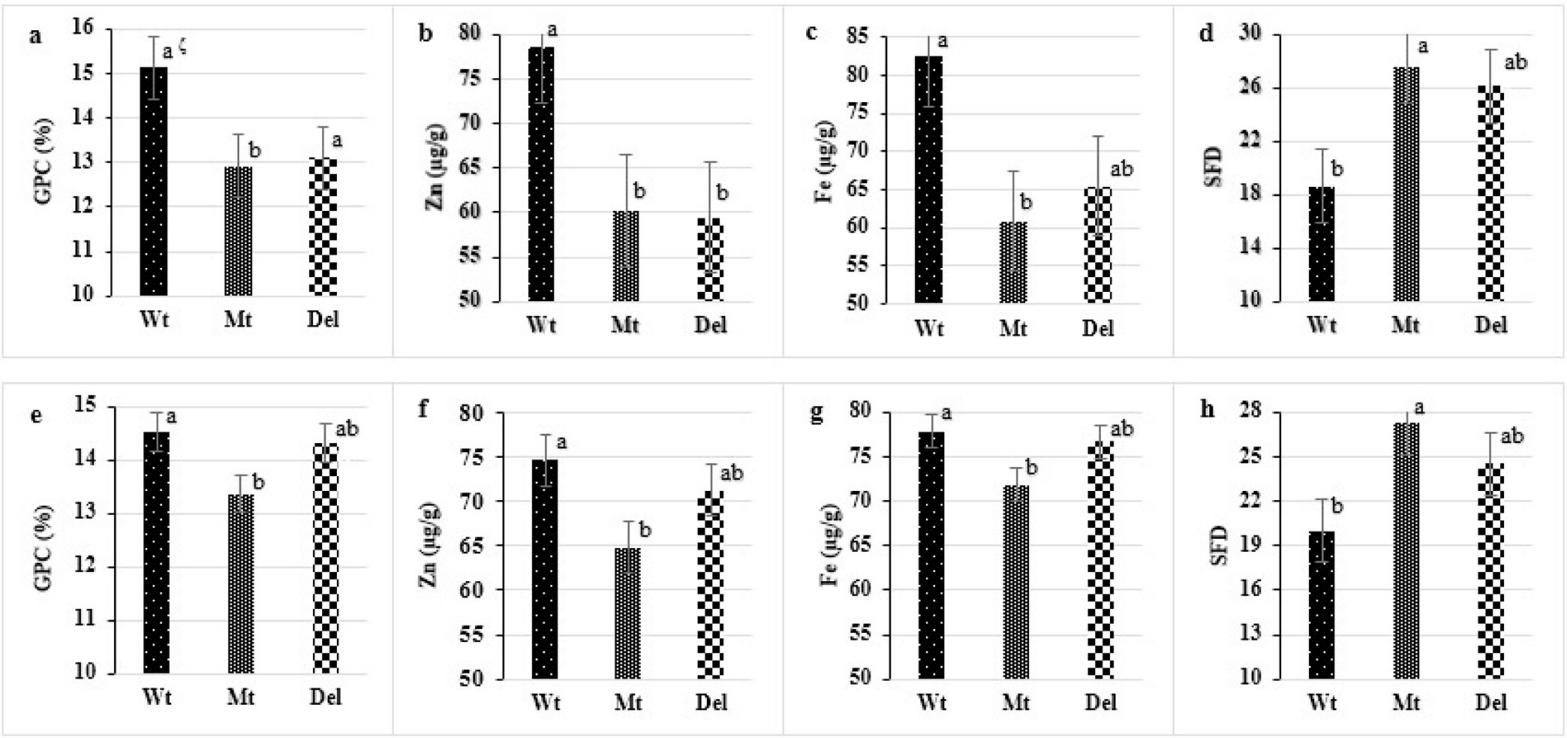
Comparison of accessions having different alleles of *NAM-B1* (wild, mutant, and deletion types) for the average over years of percent grain protein content (GPC), grain zinc (Zn) content (μg/g), grain iron (Fe) content (μg/g), and seed-filling duration (SFD). Tetraploid species (a-d). Wt; wild type (*T*. *dicoccoides*, *T*. *ispahanicum* and *T*. *dicoccum*), Mt; mutated type (*T*. *durum*, *T*. *persicum*, *T*. *polonicum*, *T*. *turgidum*, *T*. *aethiopicum* and *T*. *turanicum*), and Del; deleted type (*T*. *dicoccum*, *T*. *persicum*, *T*. *polonicum*, *T*. *turgidum*, *T*. *aethiopicum* and *T*. *turanicum*). Hexaploid species (e-h). Wt; wild type (*T*. *spelta*), Mt; mutated type (*T*. *vavilovii*, *synthetic cross* and *T*. *petropavlovskyi*), and Del; deleted type (*T*. *spelta*, *T*. *aestivum*, *T*. *compactum* and *T*. *sphaerococcum*). The bars on top of the columns represent the standard error of means. For each trait, means followed by the same letter are not significantly different, using the LSD test at a 5% probability level.

**Fig 2:**
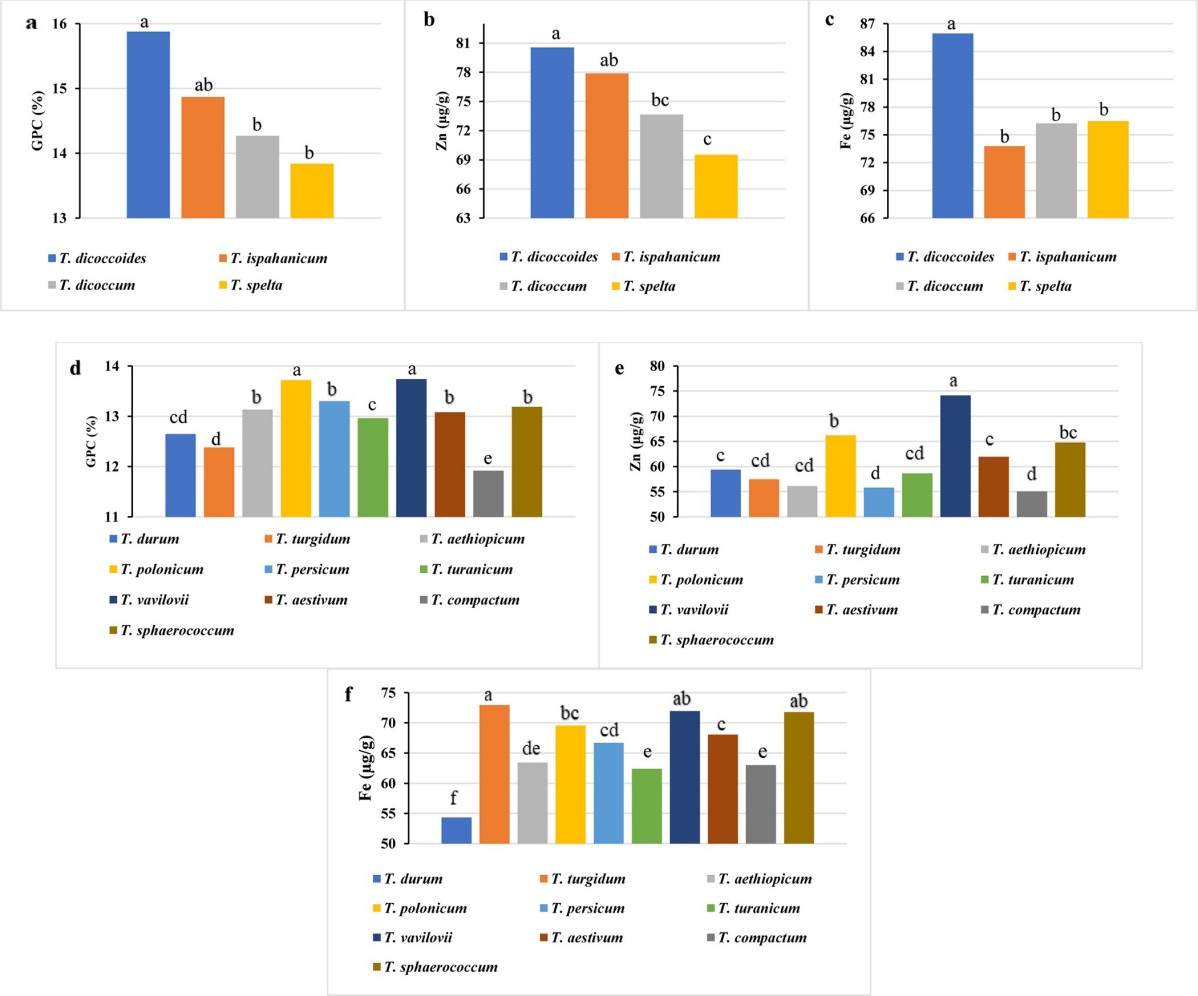
The contrast of 14 different species of tetra and hexaploid wheat for the average over years of a) percent grain protein content (GPC), grain zinc (Zn), and iron (Fe) content (μg/g). Each color represents a different species. Species with wild-type *NAM-B1* allele (a-c) and for non-functional (insertion/deletion) allele (d-f). For each trait, means followed by the same letter are not significantly different, using the LSD test at a 5% probability level.

Among the studied tetraploid species, wild emmer *T*. *dicoccoides* containing the wild-type *NAM-B1* allele showed the highest GPC values in the first experimental year (16.40%) and the second highest (15.36%) in the second year. It also had a high concentration of Zn, and Fe, with an average of 80.68 and 85.98 μg/g, respectively, over two years. Next to *T*. *dicoccoides*, the five accessions of cultivated emmer *T*. *dicoccum* possessing the wild-type *NAM-B1* allele had a significantly higher GPC, grain Zn and Fe content when compared to the accessions of the same species devoid of *NAM-B1*. This was also true when accessions having the functional type allele were compared with the accessions of all other tetraploid species (Table 1, Fig 1). Two years average of GPC for accessions of *T*. *dicoccoides*, *T*. *dicoccum*, and *T*. *ispahanicum* having the wild-type allele were 15.68, 15.10 and 14.87%, respectively. At the same time, it was 13.45% for two accessions of *T*. *dicoccum* (#8, 9), missing the allele. Also, the two years average for Zn and Fe for the two accessions of *T*. *dicoccum* missing the allele were 68.88 and 71% which was considerably less than those possessing the wild-type allele (Table 1). All nine accessions of *T*. *durum* contained the insertion mutated *NAM-B1* allele and showed a stable and slight variation for GPC over two experimental years. The average GPC over all durum accessions and two studied years was 12.65% which was considerably less than the average for accessions of *T*. *dicoccoides*, *T*. *dicoccum*, and *T*. *ispahanicum* having the wild-type allele (Fig 2). A similar trend was observed when grain Zn and Fe content of durum accessions were compared with those from *T*. *dicoccoides*, *T*. *dicoccum*, and *T*. *ispahanicum* containing the functional *NAM-B1* allele. Likewise, accessions of all other five tetraploid species with indel-type allele displayed a lower average (over two years) GPC, Zn, and Fe content compared with *T*. *dicoccoides*, *T*. *dicoccum*, and *T*. *ispahanicum* possessing the wild-type allele (Table 1).

Among the hexaploid species, *T*. *spelta* (#29) contained the wild-type allele and revealed the highest GPC values in the first experimental year (14.84%) and the second highest (14.23%) in the second year. It also had a high grain concentration of Zn, and Fe, which averaged 74.63 and 77.83 μg/g, respectively, over two studied years. It also had a higher concentration of Zn and Fe than the other accession of *T*. *spelta* (#30), which had the deletion type, and *T*. *vavilovii* (#31), having the insertion mutated-type allele. A similar trend was observed for GPC, grain Zn, and Fe content when spelt accession #29 with the wild-type *NAM-B1* allele was compared to accessions of the other hexaploid species *T*. *aestivum*, *T*. *sphaerococcum*, *T*. *compactum*, *T*. *patropavlovskyi*, and synthetic crosses containing the indel-type allele (Table 1). Another trait that negatively associated with the *NAM-B1* functional allele was the seed-filling duration (SFD). Among all 38 accessions, wild emmer *T*. *dicoccoides* possessing the wild-type allele showed the minimum SFD, both experimental years. Besides *T*. *dicoccoides*, the five accessions of *T*. *dicoccum*, one accession of *T*. *ispahanicum*, and one hexaploid accession, *T*. *spelta* (#29), having the wild-type allele resembled a lower mean for SFD compared to all other accessions containing the indel-type allele (Table 1). In terms of grain yield (GY), 1,000-kernel weight (TKW), kernel diameter (KD), and kernel length (KL), *T*. *dicoccum* accessions having wild-type allele were comparable to those containing the indel-type allele over two experimental years. However, as expected, the durum accessions had significantly higher GY, TKW, and KD than *T*. *dicoccum* accessions, whereas KL was slightly higher for *T*. *dicoccum* accessions regardless of *NAM-B1* allele status (S3 Table in S1 File).

## Discussion

Grain protein, Zn, and Fe content are three important wheat qualities crucial for human nutrition and health worldwide. Increasing these three quality components simultaneously in wheat grains by a single gene came into the picture through *NAM-B1* cloning by Uauy et al. [14]. Functional *NAM-B1* pleiotropically accelerates flag leaf senescence and causes efflux of N, Fe, Zn, and Mn from flag leaves into wheat grains, increasing protein concentrations and micronutrient levels [14, 15, 27]. The *NAM-B1* allele was first studied in wild tetraploid emmer wheat, believed to be the only source of the wild-type allele [14]. It seems that the wild-type functional allele of *NAM-B1* has been lost in durum and bread wheat during domestication and intensified by modern plant breeding [14, 22, 28]. Different researchers extensively studied the gene, confirming its significant association with grain protein, Zn, and Fe content, mostly in wild and domesticated emmer, spelt, durum, and bread wheat. The gene and its possible association with field data are less studied in a wide range of wheat species. In this study, 38 wheat accessions comprising 14 different species were field evaluated, and the *NAM-B1* gene was characterized by sequencing of the 582 first nucleotides of the gene, containing the 1-bp insertion site. The wild emmer, *T*. *dicoccoides*, accession (#1) with the highest GPC value (averaged over two experimental years), and also high grain concentration of Zn, and Fe contained the wild type *NAM-B1* allele (Table 1). *T*. *dicoccoides* is the original source of *NAM-B1* functional wild-type allele identified by Avivi [8] associated with high GPC. Among the five *T*. *dicoccum* accessions possessing the wild-type allele, four were unimproved local varieties that belonged to the central Zagros region of Iran [29], and the other one was from the CIMMYT collection. These five accessions had considerably higher GPC, grain Zn and Fe when compared with the two accessions (#8, 9) of *T*. *dicoccum* devoid of the *NAM-B1* allele. This may suggest that the pleiotropic effects of the functional *NAM-B1* allele on GPC, grain Zn and Fe are very stable in different genetic backgrounds [22, 30]. However, other minor genes may have affected the *NAM-B1*-associated traits revealed by slight variation among the wild allele genotypes. Therefore, deployment of the functional *NAM-B1* allele into wheat breeding programs has the potential of simultaneous improvement of GPC, grain Zn and Fe, in a wide range of germplasm, due to its functional stability in different genetic backgrounds [7]. This is even more promising as the increase in GPC, grain Zn and Fe caused by the functional *NAM-B1* allele has also been very stable in different environments in both tetra and hexaploid wheats. [16, 19]. The phenomenon of genotype × environment interaction is probably the number one obstacle affecting selection efficiency in a breeding program and varietal improvement [31]. Functional *NAM-B1* is also found to be associated with shorter seed-filling duration (SFD) due to an accelerated rate of senescence [14, 32]. As expected, in two experimental years, *T*. *dicoccoides* (#1) with the functional *NAM-B1* allele had the shortest SFD among all tetraploid species. Similarly, the five *T*. *dicoccum*, and one *T*. *ispahanicum*, with the wild allele, showed the same trend for SFD compared to all other tetraploid accessions containing either the deletion-type or insertion-type allele. It was reported that although the functional allele of *NAM-B1* contributes to higher grain protein and mineral content, more rapid senescence could negatively influence grain size and yield [22]. However, the adverse effect of the functional *NAM-B1* allele on grain yield has been insignificant or nonexistent in several reports [7, 16, 30]. Even with a slightly lower grain yield, better-quality wheat may have a higher market price, benefiting farmers and providing higher-quality end products for consumers [23].

The nine *T*. *durum* accessions studied were all improved cultivars with considerably higher GY, TKW, and KD than their progenitor, *T*. *dicoccum*. However, they lacked the *NAM-B1* functional allele, and all contained only the insertion muted type form. They also had lower GPC, grain Zn, and Fe compared to emmer accessions with the functional allele but almost comparable with the ones devoid of the gene. In a previous study, we crossed the emmer accession Singerd (#6 S1 Table in S1 File), having the wild type allele, with two durum accessions (#10, 11), containing the mutated type allele. In both crosses, the F1 and reciprocal backcross progenies showed higher GPC than the durum and their values were comparable to those of emmer parent without a yield penalty [33]. These findings suggest the possibility of improving GPC in durum wheat through backcross incorporation of the *NAM-B1* allele from domesticated emmer since its higher GPC can be attributed to the functional form of this gene. Thus far, most durum accessions studied for the *NAM-B1* gene possess the insertion type allele. Uauy et al. [14] reported the presence of only insertion type allele in all 57 durum lines tested. Lundström et al. [34] also found the insertion form of the allele in 15 durum wheat accessions among the 16 accessions studied.

Accessions of the less studied species of *T*. *turgidum*, *T*. *aethiopicum*, *T*. *polonicum*, *T*. *persicum*, and *T*. *turanicum* had either the deletion-type or insertion-type allele. This is the first report studying *NAM-B1* and its characterization in these species. The GPC, grain Zn, and Fe content for all these species averaged over two years were lower than their tetraploid counterparts containing the wild-type allele of *NAM-B1*. They also resembled durum and emmer accessions lacking functional alleles regarding seed-filling duration. Characterizing a higher number of accessions from these species with and without functional *NAM-B1* allele may lead to a more precise conclusion regarding *NAM-B1* role in their genetic background.

Among the hexaploid species, only one hulled wheat accession of *T*. *spelta* (#29) contained the wild-type allele of *NAM-B1*. The species of *T*. *spelta* and *T*. *vavilovii* are characterized as hexaploid hulled wheat and less subjected to breeding efforts for cultivation due to their extensive milling requirements and lower grain yield than bread wheat. The *NAM-B1* wild-type allele is reported to be more prevalent in *T*. *spelta* than in other hexaploid wheat studied [21, 22]. In our study, the spelt accession (#29) possessing the functional allele revealed higher GPC, grain Zn, and Fe concentration over two years compared to the other spelt accessions (#30), with the deletion type allele and the other hulled wheat *T*. *vavilovii* (#31), having the insertion-type one. A similar trend was observed when spelt accession #29 with the wild-type allele was compared to accessions of other hexaploid species (*T*. *aestivum*, *T*. *sphaerococcum*, *T*. *compactum*, and *T*. *patropavlovskyi*) and synthetic crosses containing the indel-type.

The functional allele of *NAM-B1* in the hexaploid genetic background has been proven to be as effective as in the tetraploid one. Brevis and Dubcovsky [16] studied the effect of the functional *NAM-B1* allele on grain yield, GPC, protein yield, and N harvest index using near-isogenic lines (>99% identical) of three tetraploid and six hexaploid wheat cultivars. They reported that protein yield was increased in the *NAM-B1* NILs of both hexaploid and tetraploid wheat, and grain protein concentration was consistently and significantly higher in the lines carrying the functional *NAM-B1* introgression relative to the controls across all genetic backgrounds and by wheat species. Similarly, by using marker-assisted selection, Kumar et al. [30] introgressed the *NAM-B1* functional allele in ten different hexaploid wheat. They reported that most *NAM-B1* introgressed lines showed considerably higher protein content than the recurrent parents lacking the allele.

## Conclusion

The field performance of 28 tetraploid wheat accessions from nine species and ten hexaploid wheat accessions from five species was evaluated for two consecutive years (2018–2020). Also, the 582 first nucleotides of the *NAM-B1* gene, containing the site of the 1-bp insertion, were investigated in this panel for possible associations between allelic variation and grain quality traits influenced by this gene. Several of these species have not been studied for *NAM-B1* previously. Among the tetraploid species, the wild-type allele of the gene was present only in *T*. *dicoccum*, *T*. *dicoccoides*, and one accession in *T*. *ispahanicum*. Fourteen tetraploid accessions contained the insertion type allele, and the gene was absent in seven remaining ones. Interestingly, all *T*. *durum* accessions studied had the insertion type allele. Based on two years of evaluation in the field, the eight tetraploid accessions possessing the wild-type *NAM-B1* allele had significantly higher GPC, grain Zn and Fe content when compared to other accessions with the insertion or deletion-type allele. Also, accessions having the wild-type allele displayed a lower mean SFD. Among the hexaploid species, five accessions had this gene, and only one of them, *T*. *spelta* (#29), contained the wild-type allele, and the rest resembled the insertion mutated type. Also, averaged over two years of evaluation, the spelt accession possessing the functional allele of the *NAM-B1* gene had higher GPC, grain Zn, and Fe concentration than the other spelt wheat accession with the deletion type allele and compared to the other hexaploid wheat accessions having the indel-type. Our results strongly support the previous findings that wheat grain protein, zinc, and iron content can be improved simultaneously by the single gene *NAM-B1*.

## Methods

### Field experiments

This study evaluated a panel of 38 wheat accessions consisting of ten hexaploids from five species and 28 tetraploids from nine species (S1 Table in S1 File) for two consecutive years in field conditions (2018–2020). These plant materials were provided by the Seed and Plant Improvement Institute of Iran (SPII), ICARDA, CIMMYT, and IPK, and landraces were provided by Isfahan University of Technology (IUT). The experiment was conducted at Isfahan University of Technology’s Research Farm (32°32 N, 51°23 E, 1630 m altitude) with a clay loam soil texture (pH of 7.5) and average annual precipitation and temperature of 140 mm and 15°C, respectively (S2 Table in S1 File). A randomized complete block design with two replications was used in the experiments. Each plot consisted of two rows, each 100 cm long and 20 cm apart (0.40 m^2^). The traits were measured on each plot separately. Phenological traits were measured according to Zadoks et al. [35] scales. Days to 50% heading (DH, stage 55) were recorded from the sowing date up to the emergence of 50% of the spikes. Days to 50% anthesis (DA, stage 61) were noted from the sowing date till 50% of plants completed anthesis. Days to physiological maturity (PM, stage 94) were recorded from the sowing date to the physiological maturity. Seed-fill duration (SFD) was calculated by subtracting PM from DA. Grain yield per plot (GY g/m^2^), 1,000 kernel weight (TKW g), kernel diameter (KD mm), and kernel length (KL mm) were also measured for each plot. Grain protein content (GPC %) was determined using near-infrared spectroscopy (International Association for Cereal Science and Technology (ICC) standard method 159, Vienna, Austria). Grain Zn and Fe contents were determined by ashing 0.2 g grain samples at 550°C for three hours and extraction with 10 ml 2N HCl. Rayleigh WFX-210 flame atomic absorption spectrometer was used to measure Zn and Fe concentrations in extracts. All grain micronutrient concentrations were expressed on a dry weight basis. SAS 9.4 (SAS Institute, Cary, NC, USA) was used to analyze the data for mean comparisons, and Excel was used to make figures.

### Genotyping

#### DNA extraction and PCR amplification of *NAM-B1*

**Fig 3:**
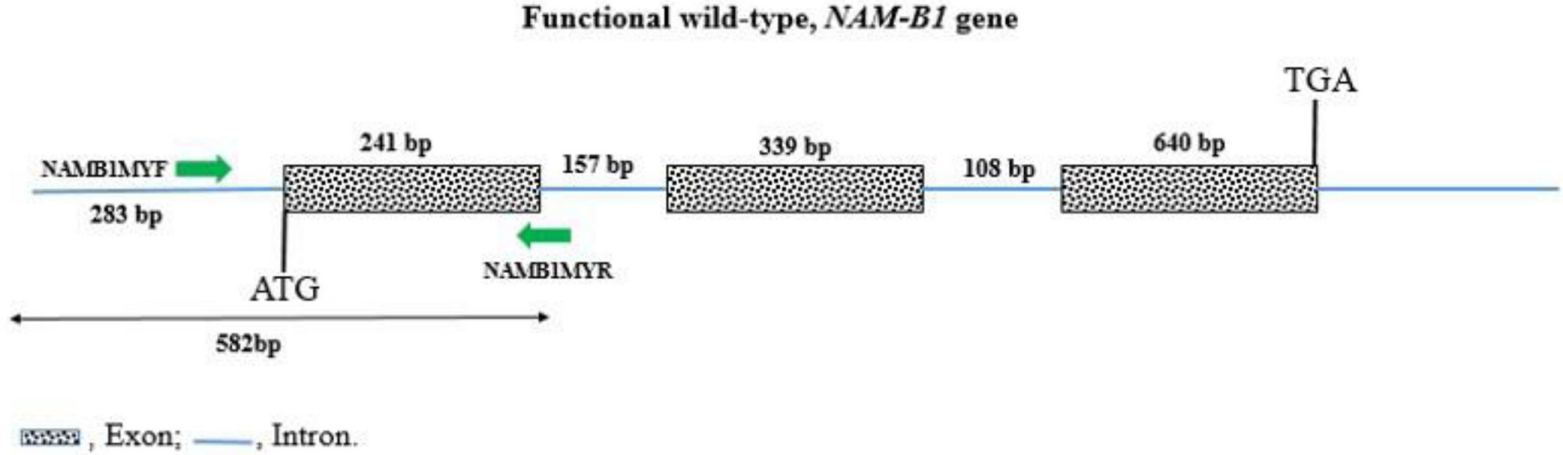
Diagram showing the functional wild-type of *NAM-B1* and the 582 bp amplified and sequenced in studied wheat species.

Seeds of the 38 wheat accessions were treated with fungicide and sown separately in plastic boxes containing moist tissue and kept in a controlled growth room. After seven days, leaves from 10 seedlings of each accession were collected and flash-frozen into an Eppendorf tube. Total DNA was isolated using a Mag-Bind^®^ Plant DNA DS kit (Omega Bio-Tek, Norcross, USA) following the manufacturer’s instructions. Frozen shoots were mechanically ground before DNA extraction to homogenize the plant tissue. DNA purity and concentration were tested using a NanoDrop 2000 (Thermo Scientific, Waltham, USA). Agarose gel was used for the verification of integrity of isolated DNA. For PCR, the extracted genomic DNA was diluted with sterilized distilled water to 2.5 ng μL^-1^. The PCR amplification of the 582 first nucleotides of the *NAM-B1* gene (Fig 3), containing the site of the 1-bp insertion, was carried out as in Yang et al. [24] to genotype the 38 wheat accessions for the three described *NAM-B1* alleles (wild-type, 1-bp insertion, and deletion). A pair of specific primers for *NAM-B1* [24], NAMB1MYF (CCCCGGGCTAGGTACAAAGGT), and NAMB1MYR (AATTTGCGGCGCTTGATAAAG) was used for amplifying and screening the gene.

All PCRs were conducted in 15 μl volumes containing 5μl of genomic DNA (2.5 ng μl^–1^) and 10 μl of master mix containing: 0.06μl of *Dream Taq* polymerase (5u/um), 1.5μl of 10x Green buffer, 0.3μl of 10 mM dNTPs, 0.15μl of 50 mM MgCl_2_, 6.79μl Milli-Q Water, and 0.6μl of each reverse and forward primers. The PCR reaction was programmed at 94°C for 3 min to denature the DNA, followed by 35 cycles of 94°C for 30s, 58°C for 30s, and 72°C for 1 min, with a final extension step at 72°C for 5 min. The PCR-amplified products were separated on 1.0% agarose gel. The amplification was consistently repeated two times for all entries.

#### Sequence analysis

The 582 bp *NAM-B1* PCR product was gel-purified or cleaned-up using the AMPure XP beads (PN B37419AA Beckman Coulter, Inc. 250 S. Kraemer Blvd. Brea, CA 92821, USA) before sequencing. PCR amplicons were sequenced at Eurofins Genomics (GmbH, Anzinger, Ebersberg, Germany). Sequences of the *NAM-B1* amplicon were analyzed using Snap Gene^®^ Viewer version 5.3.2 and ApE-A plasmid Editor version 3.0.6 to discriminate between the wild-type and the 1-bp insertion alleles. The sequences of the *NAM-B1* allele from the 26 accessions were compared with the previously reported wild-type allele DQ869673.1 [14].

## Supporting information

S1 File Information on 38 wheat accessions from different species used in the study. (DOCX)

## Abbreviations

GPC: Grain protein content
Indel-type: Insertion-deletion type
*NAM-B1*: No Apical Meristem-B1
(SFD): Seed-filling duration
