# Supplementary material for: Characterization of 14 Triticum species for the NAM-B1 gene and its associated traits
Source: PLoS One. 2023 Aug 22;18(8):e0287798. doi: 10.1371/journal.pone.0287798 (PMC10443865; doi:10.1371/journal.pone.0287798)
Supplement: S1 File — (DOCX) [file pone.0287798.s001.docx]

| **No.** | **Species/Name** | **Ploidy levels** | **Origin/Code** | **No.** | | **Species/Name** | **Ploidy levels** | **Origin/Code** |
| --- | --- | --- | --- | --- | --- | --- | --- | --- |
| 1 | *T. dicoccoides* | 4x | Iran | 20 | | *T. turgidum* | 4x | IPK, TRI9652 |
| 2 | *T. ispahanicum* | 4x | IPK^1^,TRI 6177 | 21 | | *T. aethiopicum* | 4x | IPK, TRI 15657 |
| 3 | *T. dicoccum* (Khoyghan )¥ | 4x | Iran | 22 | | *T. aethiopicum* | 4x | IPK, TRI 15593 |
| 4 | *T. dicoccum* (Ozonbelagh)¥ | 4x | Iran | 23 | | *T. polonicum* | 4x | IPK, TRI 1896 |
| 5 | *T. dicoccum* (Zarneh)¥ | 4x | Iran | 24 | | *T. polonicum* | 4x | Iran |
| 6 | *T. dicoccum* (Singerd)¥ | 4x | Iran | 25 | | *T. persicum* | 4x | IPK, TRI 9535 |
| 7 | *T. dicoccum* | 4x | CIMMYT^2^, 49655 | 26 | | *T. turanicum* | 4x | Iran |
| 8 | *T. dicoccum* | 4x | IPK,TRI 19124 | 27 | *T. turanicum* | | 4x | IPK,TRI 17606 |
| 9 | *T. dicoccum* | 4x | IPK,TRI 17619 | 28 | *T. spelta* | | 4x | Iran |
| 10 | *T. durum* (Yavaros) | 4x | CIMMYT | 29 | *T. spelta* | | 6x | IPK,TRI 3429 |
| 11 | *T. durum* (Shabrang) | 4x | CIMMYT | 30 | *T. vavilovii* | | 6x | Iran |
| 12 | *T. durum* (Karkheh) | 4x | ICARDA^3^ | 31 | *T. aestivum* | | 6x | IPK, TRI 11554 |
| 13 | *T. durum* (Behrang) | 4x | CIMMYT | 32 | *T. aestivum* (Chines Spring) | | 6x | Iran |
| 14 | *T. durum* (Saji) | 4x | ICARDA | 33 | | *Synthetic cross*^4^ | 6x | Iran |
| 15 | *T. durum* (Shwa) | 4x | ICARDA | 34 | | *Synthetic cross* | 6x | CIMMYT, BW 39452 |
| 16 | *T. durum* (Ariya) | 4x | ICARDA | 35 | | *T. compactum* | 6x | CIMMYT, BW 31696 |
| 17 | *T. durum* (Dena) | 4x | CIMMYT | 36 | | *T. petropavlovski* | 6x | Iran |
| 18 | *T. durum* (Langdon) | 4x | Iran | 37 | | *T. sphaerococcum* | 6x | IPK,TRI 12908 |
| 19 | *T. turgidum* | 4x | Iran | 38 | | *T. sphaerococcum* | 6x | IPK,TRI 18664 |
|  |  |  |  |  | |  |  |  |

**Table S1 Information on 38 wheat accessions from different species used in the study**

^1^ Leibniz Institute of Plant Genetics and Crop Plant Research

^2^ International Maize and Wheat Improvement Center

^3^ International Center for Agricultural Research in the Dry Areas

^4^ Synthetic cross obtained from a cross between *T. turgidum* ssp. *durum* with *Aegilops tauschii*

^¥,^ Local variety (Landrace), from the central Zagros region of Iran, ^¥^, SNP, Single nucleotide polymorphism at position +11

**Table S2 Physico-chemical properties of soil in experimental site (0-60 cm soil depth)**

| **Soil particles (%)** | | | **Soil texture** | **EC**  **(ds.m^-1^)** | **PH** | **Cu**  **(mg/kg)** | **Mn**  **(mg/kg)** | **Fe**  **(mg/kg)** | **Zn**  **(mg/kg)** | **P**  **(mg/kg)** | **K**  **(mg/kg)** | **T.N**  **(%)** | **Soil depth**  **(cm)** | | **Year** |
| --- | --- | --- | --- | --- | --- | --- | --- | --- | --- | --- | --- | --- | --- | --- | --- |
| **Sand** | **Clay** | **Silt** |  |  |  |  |  |  |  |  |  |  |  |  |  |
| 40 | 30 | 30 | Clay-Loam | 3.9 | 7.7 | 0.74 | 11.46 | 4.15 | 1.16 | 31.38 | 526.6 | 0.06 | 0-30 | 2018-2019 | |
| 36.67 | 33.33 | 30 |  | 2.16 | 7.53 | 0.61 | 6.02 | 4.03 | 0.69 | 19.13 | 483.1 | 0.03 | 30-60 |  |  |
| 41 | 25 | 34 | Loam | 2.1 | 7.70 | 1.3 | 20.1 | 14.9 | 2.5 | 38.6 | 495 | 0.08 | 0-30 | 2019-2020 | |
| 40 | 26 | 34 | Loam | 1.6 | 7.95 | 0.85 | 7.5 | 10.1 | 0.63 | 6.7 | 300 | 0.03 | 30-60 |  | |

Total nitrogen (T.N); Electrical conductivity (EC).

**Table S3 Mean comparison of studied traits for 38 genotypes in each year of evaluation (2018-2020).**

| **No.** | **Species** | **PL^δ^** | ***NAM-B1*** | **SNP**^¥^ | **GY-^1yr^** | **GY-^2yr^** | **TKW-^1yr^** | **TKW-^2yr^** | **KL-^1yr^** | **KL-^2yr^** | **KD-^1yr^** | **KD-^2yr^** |
| --- | --- | --- | --- | --- | --- | --- | --- | --- | --- | --- | --- | --- |
| 1 | *T. dicoccoides* | 4x | Wild type | C | 336.25q ^ζ^ | 410pq | 29.30lmn | 29.57pq | 8.85ab | 8.50ab | 2.44r | 2.47l |
| 2 | *T. ispahanicum* | 4x | Wild type | C | 528.75m-p | 447.5pq | 26.67j-m | 28.53q | 8.23bc | 8.67a | 2.83pq | 2.78f-j |
| 3 | *T. dicoccum* | 4x | Wild type | C | 581.25l-o | 508.10nop | 30.83j-m | 30.76opq | 7.80cde | 7.55e-i | 2.84opq | 2.70g-l |
| 4 | *T. dicoccum* | 4x | Wild type | C | 598.75lmn | 460.60opq | 22.32n | 30.84n-q | 7.39d-j | 7.22g-l | 2.81opq | 2.73g-k |
| 5 | *T. dicoccum* | 4x | Wild type | C | 468.75n-p | 513.10nop | 32.86h-m | 31.70m-q | 7.91cde | 7.40f-j | 2.92m-q | 2.66h-l |
| 6 | *T. dicoccum* | 4x | Wild type | C | 567.75l-o | 552.50m-q | 26.87mn | 32.29l-q | 7.90cde | 8.24abc | 2.85opq | 2.58i-l |
| 7 | *T. dicoccum* | 4x | Wild type | C | 453.75opq | 742.50i-m | 31.11i-m | 28.95q | 6.93h-n | 7.30f-k | 2.80q | 2.52kl |
| 8 | *T. dicoccum* | 4x | Absent | - | 577.50l-o | 552.50m-p | 27.80mn | 32.60k-q | 7.32e-k | 7.94cde | 3.09h-m | 2.88e-h |
| 9 | *T. dicoccum* | 4x | Absent | - | 387.50q | 460k-o | 31.40i-m | 35.54j-p | 7.46d-j | 8.09bcd | 3k-p | 2.81f-j |
| 10 | *T. durum* | 4x | Mutated | T | 892.50d-q | 900.60f-j | 44.78b-f | 37.79f-m | 6.90i-n | 6.73l-p | 3.31b-g | 3.10a-e |
| 11 | *T. durum* | 4x | Mutated | T | 938.75d | 901.30f-j | 42.98b-g | 43.98b-f | 7.30e-k | 7.10h-m | 3.35b-e | 3.13a-e |
| 12 | *T. durum* | 4x | Mutated | T | 921.25de | 921.30e-j | 45.87a-e | 36.60i-o | 7.06g-l | 6.79k-p | 3.37a-e | 2.94c-f |
| 13 | *T. durum* | 4x | Mutated | T | 737.50h-k | 875.60g-j | 47.68abc | 45.88a-e | 7.45d-j | 7.54e-i | 3.40a-d | 3.29a |
| 14 | *T. durum* | 4x | Mutated | T | 916.25def | 960d-i | 36.19g-l | 42.66b-i | 6.69k-o | 6.94j-m | 3.19e-i | 3.26ab |
| 15 | *T. durum* | 4x | Mutated | T | 740h-k | 1152.50a-d | 44.89b-f | 41.15d-j | 7.71c-f | 7.26g-l | 3.28c-g | 3.16abc |
| 16 | *T. durum* | 4x | Mutated | T | 1181.75bc | 887.50f-j | 53.19a | 38.64f-l | 7.31e-k | 7.07h-m | 3.47ab | 3.16abc |
| 17 | *T. durum* | 4x | Mutated | T | 872.50d-h | 878.10g-j | 40.06c-h | 37.46g-m | 6.63l-o | 6.79k-d | 3.33b-f | 3.18abc |
| 18 | *T. durum* | 4x | Mutated | T | 916.25def | 1152.50a-d | 38.13e-j | 35.39j-p | 7.14f-l | 6.76l-p | 3.03j-o | 2.83f-i |
| 19 | *T. turgidum* | 4x | Absent | - | 1176.25bc | 1040c-h | 39.99c-h | 44.03b-f | 6.87j-n | 6.92j-o | 3.15f-k | 3.17abc |
| 20 | *T. turgidum* | 4x | Mutated | T | 793.75e-i | 591.3l-o | 38.53d-j | 43.37b-g | 6.31no | 5.94r | 3.39a-d | 3.15a-d |
| 21 | *T. aethiopicum* | 4x | Absent | - | 1305ab | 1275ab | 38.68d-i | 41.37c-j | 7.35e-j | 6.89j-o | 3.06i-n | 2.81f-j |
| 22 | *T. aethiopicum* | 4x | Mutated | T | 836.25d-h | 1062.5b-g | 47.63abc | 51.35a | 7.38d-j | 7.06i-m | 3.37a-d | 3.04a-f |
| 23 | *T. polonicum* | 4x | Mutated | T | 177.50r | 400pq | 37.57f-k | 41.96c-i | 7.63c-g | 7.78c-f | 3k-p | 2.95c-g |
| 24 | *T. polonicum* | 4x | Absent | - | 648.75j-m | 1102.5b-f | 37.78f-k | 39.35f-j | 6.35mno | 6.52n-q | 3.31b-g | 3.03b-f |
| 25 | *T. persicum* | 4x | Mutated | T | 415pq | 635k-o | 31.66i-m | 30.76opq | 6.36mno | 6.09qr | 2.89n-q | 2.57jkl |
| 26 | *T. persicum* | 4x | Absent | - | 618.75klm | 715j-n | 32.39h-m | 32.96k-q | 6.22o | 6.06qr | 2.96l-q | 2.72g-l |
| 27 | *T. turanicum* | 4x | Mutated | T | 652.50j-m | 827.5h-j | 48.62ab | 47.62abc | 9.28a | 7.67d-g | 3.41abc | 3.25ab |
| 28 | *T. turanicum* | 4x | Absent | - | 1115c | 1135a-e | 40.81c-g | 50.63a | 8cd | 7.59d-h | 3.39a-d | 3.26ab |
| 29 | *T. spelta* | 6x | Wild type | C | 373.75q | 410pq | 36.64g-l | 37.76f-m | 7.39d-j | 7.78c-f | 3.05i-n | 3.02b-f |
| 30 | *T. spelta* | 6x | Absent | - | 863d-h | 797.5i-l | 37.51f-k | 37.72h-o | 7.51d-i | 7.05i-m | 3.42abc | 3.09a-e |
| 31 | *T. vavilovii* | 6x | Mutated | T | 470n-p | 930e-j | 39.56d-h | 42.71b-i | 7.52d-i | 6.82k-o | 3.38a-d | 3.17abc |
| 32 | *T. aestivum* | 6x | Absent | - | 1381.25a | 1201abc | 42.02b-f | 46.76a-d | 6.97h-l | 6.90j-o | 3.32b-f | 3.12a-e |
| 33 | *T. aestivum* | 6x | Absent | - | 935d | 1262.50ab | 39.68d-h | 37.12g-n | 6.13o | 6.36pqr | 3.13g-l | 3.18abc |
| 34 | *synthetic cross* | 6x | Mutated | T | 883.75f-j | 1241.30abc | 43.83b-g | 46.76a-d | 6.33mno | 6.14qr | 3.42abc | 3.23ab |
| 35 | *synthetic cross* | 6x | Mutated | T | 855d-h | 1345a | 45.08b-f | 42.70b-i | 6.54l-o | 6.68m-p | 3.27c-h | 3.25ab |
| 36 | *T. compactum* | 6x | Absent | - | 1116.25c | 1105b-f | 38.26e-j | 38.87f-k | 6.38mno | 6.41o-r | 3.27c-h | 3.03a-f |
| 37 | *T. petropavlovskyi* | 6x | Mutated | T | 685i-l | 832.5h-j | 46.06a-d | 48.76ab | 7.55d-h | 7.94cde | 3.42abc | 3.23ab |
| 38 | *T. Sphaerococcum* | 6x | Absent | - | 830d-h | 822.5h-j | 30.29klm | 32.73k-q | 5.19p | 5.21s | 3.22d-i | 2.90d-h |
|  | LSD (0.05) |  |  |  | 136.10 | 220.81 | 7.78 | 6.35 | 0.63 | 0.52 | 0.18 | 0.25 |

GY, Grain yield (g/m^2^); TKW, 1,000 kernel weight (g). KL, kernel length (mm); KD, kernel diameter (mm). ^ζ^, For each column, means followed by the same letter are not significantly different, using

the LSD test at a 5% probability level.

**^δ,^** Ploidy Level**;** ^¥^, SNP, Single nucleotide polymorphism at position +11

**Table S3 Continued**

| **No.** | **Species** | **LP** | ***NAM-B1*** | **SNP**^¥^ | **DH-^1yr^** | **DH-^2yr^** | **DA-^1yr^** | **DA-^2yr^** | **PM-^1yr^** | **PM-^2yr^** |
| --- | --- | --- | --- | --- | --- | --- | --- | --- | --- | --- |
| 1 | *T. dicoccoides* | 4x | Wild type | C | 184.50f-i ^ζ^ | 178.50f-i | 188lmn | 184j-n | 209.50n | 193o |
| 2 | *T. ispahanicum* | 4x | Wild type | C | 191.50abc | 193.50a | 198cd | 197.50a | 224gh | 210cde |
| 3 | *T. dicoccum* | 4x | Wild type | C | 191abc | 187bcd | 197cde | 194a-d | 223g-j | 207d-h |
| 4 | *T. dicoccum* | 4x | Wild type | C | 191abc | 184c-f | 198.50c | 192c-f | 222.50g-k | 206e-i |
| 5 | *T. dicoccum* | 4x | Wild type | C | 190a-d | 185.50cde | 196c-f | 193.50b-e | 220.50j-m | 206e-i |
| 6 | *T. dicoccum* | 4x | Wild type | C | 190a-d | 184c-f | 197cde | 192c-f | 222h-k | 205.50f-j |
| 7 | *T. dicoccum* | 4x | Wild type | C | 189b-f | 183.50c-g | 195.50d-g | 190.50d-g | 222h-k | 204h-l |
| 8 | *T. dicoccum* | 4x | Absent | - | 188.50c-f | 191.50ab | 196.50cde | 197ab | 229de | 216ab |
| 9 | *T. dicoccum* | 4x | Absent | - | 191.50abc | 193.50a | 197.50cde | 197ab | 230de | 216.50a |
| 10 | *T. durum* | 4x | Mutated | T | 178.50k-o | 171.50jkl | 190jkl | 183.50k-n | 223.50ghi | 203.50h-l |
| 11 | *T. durum* | 4x | Mutated | T | 176m-p | 171.50jkl | 188.50k-n | 181.50m-q | 220klm | 204.50h-k |
| 12 | *T. durum* | 4x | Mutated | T | 175.50nop | 170kl | 189.50klm | 184j-n | 222.50g-k | 204.50h-k |
| 13 | *T. durum* | 4x | Mutated | T | 176m-p | 172jk | 187mn | 182.50k-o | 222.50g-k | 204h-l |
| 14 | *T. durum* | 4x | Mutated | T | 175op | 170kl | 186n | 182l-p | 222h-k | 204h-l |
| 15 | *T. durum* | 4x | Mutated | T | 175op | 173ijk | 190jkl | 184j-n | 221i-l | 205.50f-j |
| 16 | *T. durum* | 4x | Mutated | T | 178.50k-o | 170kl | 187mn | 185j-m | 222.50g-k | 205.5-f-j |
| 17 | *T. durum* | 4x | Mutated | T | 176m-p | 173ijk | 188.50k-n | 183k-o | 221.50h-k | 206e-i |
| 18 | *T. durum* | 4x | Mutated | T | 182h-l | 173.50ijk | 191ijk | 186h-k | 225fg | 207d-h |
| 19 | *T. turgidum* | 4x | Absent | - | 194.50a | 180.50e-h | 197.50cde | 189f-i | 227.50ef | 209c-g |
| 20 | *T. turgidum* | 4x | Mutated | T | 190.50abc | 182.50d-g | 193ghi | 189.50fgh | 223.50ghi | 211cd |
| 21 | *T. aethiopicum* | 4x | Absent | - | 176.50m-p | 170kl | 187.50lmn | 178qrs | 223g-j | 204.50h-j |
| 22 | *T. aethiopicum* | 4x | Mutated | T | 178k-p | 171.50jkl | 187.50lmn | 180.50n-r | 220.50j-m | 206e-i |
| 23 | *T. polonicum* | 4x | Mutated | T | 190a-d | 187bcd | 207.50b | 195.50abc | 257.50a | 215.50ab |
| 24 | *T. polonicum* | 4x | Absent | - | 189.50b-e | 188.50abc | 198cd | 194a-d | 229de | 212.50abc |
| 25 | *T. persicum* | 4x | Mutated | T | 180i-n | 176.50hij | 189.50klm | 182.50k-o | 218.50lm | 203.50h-k |
| 26 | *T. persicum* | 4x | Absent | - | 180.50h-m | 178.50f-i | 188lmn | 185j-m | 218m | 205g-j |
| 27 | *T. turanicum* | 4x | Mutated | T | 185.50d-f | 178ghi | 191ijk | 187.50g-m | 224gh | 207d-h |
| 28 | *T. turanicum* | 4x | Absent | - | 173.50p | 166l | 190jkl | 176.50j | 221.50h-k | 196no |
| 29 | *T. spelta* | 6x | Wild type | C | 193.50ab | 181e-h | 224a | 195.50abc | 250b | 209.50c-f |
| 30 | *T. spelta* | 6x | Absent | - | 189.50b-e | 183.50c-g | 193.50f-i | 192c-f | 231cd | 212bc |
| 31 | *T. vavilovii* | 6x | Mutated | T | 190a-d | 178.50f-i | 195.50d-g | 185.50i-l | 233c | 203h-l |
| 32 | *T. aestivum* | 6x | Absent | - | 177.50l-p | 168.50kl | 195.50d-g | 179.50o-s | 228e | 202i-m |
| 33 | *T. aestivum* | 6x | Absent | - | 175op | 169kl | 183o | 176j | 220.50j-m | 198.50mn |
| 34 | *synthetic cross* | 6x | Mutated | T | 183.50h-j | 168.50kl | 188lmn | 176.50j | 222h-k | 200.50klm |
| 35 | *synthetic cross* | 6x | Mutated | T | 180i-n | 169.50kl | 188lmn | 177rs | 222.50g-k | 200lmn |
| 36 | *T. compactum* | 6x | Absent | - | 183.50j-j | 185cde | 192.50hij | 192c-f | 223g-j | 211cd |
| 37 | *T. petropavlovskyi* | 6x | Mutated | T | 188.50c-f | 184.50cde | 192.50hij | 192c-f | 222.50g-k | 211cd |
| 38 | *T. Sphaerococcum* | 6x | Absent | - | 182.50h-k | 169.50kl | 191ijk | 178.50p-s | 222.50g-k | 201.50j-m |
|  | LSD (0.05) |  |  |  | 4.80 | 5.53 | 2.59 | 3.97 | 2.71 | 4.02 |

DH, day to heading; DA, day to anthesis; PM, day to physiological maturity. ^ζ^, For each column, means followed by the same letter are not significantly

different, using the LSD test at a 5% probability level.

**^δ,^** Ploidy Level**;** ^¥^, SNP, Single nucleotide polymorphism at position +11
